# Stress, Anxiety, and Self-Efficacy in Hypertension: Evidence from a Romanian Case—Control Study

**DOI:** 10.3390/diseases13110373

**Published:** 2025-11-13

**Authors:** Lucia Bubulac, Mirela Zivari, Irina Anca Eremia, Constantin Erena, Consuela-Mădălina Gheorghe, Iuliana-Raluca Gheorghe, Viorica Tudor, Claudia Florina Bogdan-Andreescu, Emin Cadar, Cristina-Crenguța Albu

**Affiliations:** 1 Department of Family Medicine, Faculty of Medicine, “Carol Davila” University of Medicine and Pharmacy, 020021 Bucharest, Romania; lucia.bubulac@umfcd.ro (L.B.); irina.eremia@umfcd.ro (I.A.E.); 2 Department of Psychology and Educational Sciences, Faculty of Psychology, Ecological University of Bucharest, 061341 Bucharest, Romania; mirela.zivari@ueb.education.ro; 3 Department of Psychology, Emergency University Hospital, 050098 Bucharest, Romania; 4 Internal Medicine Department, Bucharest Emergency Clinical Hospital, Faculty of Medicine, “Carol Davila” University of Medicine and Pharmacy, 020021 Bucharest, Romania; constantin.erena@umfcd.ro; 5 Department of Marketing and Medical Technology, Faculty of Medicine, “Carol Davila” University of Medicine and Pharmacy, 020021 Bucharest, Romania; consuela.gheorghe@umfcd.ro; 6 Integrated Outpatient Department of Obstetrics and Gynecology, “Prof. Dr. Agrippa Ionescu” Clinical Emergency Hospital, 011356 Bucharest, Romania; 7 Department of Speciality Disciplines, “Titu Maiorescu” University, 031593 Bucharest, Romania; claudia.andreescu@prof.utm.ro; 8 Faculty of Pharmacy, “Ovidius” University, 900470 Constanța, Romania; emin.cadar@365.univ-ovidius.ro; 9 Department of Genetics, Faculty of Dentistry, “Carol Davila” University of Medicine and Pharmacy, 020021 Bucharest, Romania; cristina.albu@umfcd.ro

**Keywords:** anxiety, hypertension, psychological distress, self-efficacy, stress vulnerability

## Abstract

**Background:** Hypertension and psychological distress often coexist, though evidence from Eastern Europe is still limited. Stress, anxiety, and self-efficacy influence blood pressure control and treatment adherence. Their effect on hypertension prevention and treatment has not been systematically evaluated in Romania. **Aim:** This study evaluated the associations between stress, anxiety, and self-efficacy in Romanian adults with and without hypertension to identify modifiable psychological factors relevant for integrated cardiovascular management. **Methods:** A retrospective case–control study was conducted among 215 adults, including individuals with hypertension and normotensive controls. Participants completed validated questionnaires assessing stress vulnerability, perceived stress, state and trait anxiety, self-efficacy, and Type A behavior, together with demographic and occupational data. **Results:** Hypertensive participants reported higher stress vulnerability, perceived stress, and anxiety, as well as lower self-efficacy, compared with controls. Type A behavior showed no association with hypertension. These differences remained consistent after accounting for demographic characteristics. **Conclusions:** Hypertension in Romanian adults is associated with a distinct psycho-emotional profile characterized by elevated stress and anxiety and reduced self-efficacy. Type A personality showed no association. The results emphasize the importance of recognizing and addressing modifiable psychological determinants in hypertension care. Integrating psychosocial assessment with personalized interventions, including mindfulness-based approaches, digital health support, and nurse-led telemonitoring, could improve treatment adherence, reduce emotional burden, and contribute to overall cardiovascular health. This region-specific evidence supports expanding hypertension management to include psychological care alongside standard medical approaches.

## 1. Introduction

### 1.1. Global Burden of Hypertension

Hypertension (HTN) remains one of the most important global health challenges of the 21st century. Despite advances in pharmacological therapy and preventive strategies, it continues to be the leading modifiable risk factor for premature death and disability worldwide [1]. Between 1990 and 2019, the number of adults aged 30–79 years with HTN nearly doubled, from ~648 million to 1.28 billion, with two-thirds of cases in low- and middle-income countries (LMICs) [1]. In parallel, the mortality burden rose from 7.2 million deaths in 1990 to ~10.9 million in 2021, mainly due to ischemic heart disease, stroke, and heart failure [2,3]. Eastern Europe records some of the highest rates of uncontrolled HTN, and in Romania, for 2023, the WHO estimates approximately 6.6 million adults (46% of the population aged 30–79) with HTN, causing >90,000 annual deaths [4]. These data highlight the need for new and multidisciplinary strategies that extend beyond traditional cardiovascular management.

The 2024 ESC Guidelines introduce the concept of “elevated blood pressure” (office blood pressure 120–139/70–89 mmHg) while maintaining hypertension at ≥140/90 mmHg. They emphasize the importance of out-of-office blood pressure monitoring for diagnosis and follow-up and recommend treating most adults to a target range of 120–129/70–79 mmHg, if tolerated. The guidelines place a greater focus on implementation, individualized risk-based management, and psychosocial factors that influence adherence and blood pressure control [5].

### 1.2. Mental Health Disorders: A Parallel Epidemic

Mental disorders represent a major and growing contributor to the global burden of disease. According to the Global Burden of Disease (GBD) 2019 analysis, nearly one in eight people worldwide—approximately 970 million individuals—were living with a mental disorder, the most prevalent being anxiety (301 million cases) and depression (280 million) [6]. Recent updates from the World Health Organization (WHO) confirm a continuing upward trend, particularly in low- and middle-income countries where access to mental healthcare remains limited [7].

The COVID-19 pandemic intensified this public health challenge, heightening stress, anxiety, and depressive symptoms in all age groups [8]. Lockdowns, social isolation, job insecurity, and prolonged uncertainty contributed to widespread psychological distress, which increased the risk of chronic conditions, including hypertension and cardiovascular disease [9,10,11]. Even after the pandemic officially ended, global monitoring reports indicated that high levels of stress and anxiety continued, reflecting long-term psychosocial effects and continued socioeconomic instability [12].

At a biological level, chronic psychological stress triggers neuroendocrine dysregulation by activating the hypothalamic–pituitary–adrenal (HPA) axis and the sympathetic nervous system (SNS), creating downstream neuroendocrine and inflammatory effects that promote cardiovascular dysfunction [13,14].

Moreover, stress-related psychiatric disorders are increasingly understood as systemic conditions with immune, metabolic, and vascular implications rather than purely psychological phenomena [15].

From a sociocultural perspective, ongoing geopolitical tension, economic recession, and accelerated lifestyle changes have compounded psychosocial stress in many European regions [16]. Eastern Europe, in particular, has reported some of the highest rates of psychological distress in the WHO European Region, coupled with inadequate access to integrated mental health services [17]. In Romania, national health surveys indicate that anxiety and depressive symptoms affect a growing proportion of adults, are often undiagnosed or untreated, and frequently coexist with hypertension [18].

These converging epidemiological and pathophysiological trends emphasize the need for region-specific studies exploring how psychosocial stressors interact with biological risk factors to modify hypertension outcomes [19]. Understanding these interconnections within the Romanian context may facilitate more effective, culturally adapted prevention and treatment strategies that link cardiovascular and mental healthcare.

### 1.3. Bidirectional Relationship Between Hypertension and Mental Health

Evidence consistently shows that the relationship between HTN and mental health disorders is bidirectional. Individuals with HTN are at increased risk of developing anxiety and depression, whereas those with psychiatric disorders have a higher likelihood of incident HTN [20,21,22,23,24]. The coexistence of HTN and depression is particularly harmful, being associated with greater all-cause and cardiovascular mortality [25]. Moreover, longitudinal studies demonstrate that depression predicts incident HTN, conferring a 54% higher risk compared with individuals without depression [26].

### 1.4. Pathophysiological Underpinnings

The link between mental health disorders and HTN involves behavioral, neuroendocrine, and inflammatory mechanisms. Risk behavioral factors, like smoking, alcohol, poor diet, sedentarism, and low adherence to treatment, contribute to HTN progression [27,28,29,30]. Chronic psychosocial stress activates the sympathetic nervous system (SNS) and the hypothalamic–pituitary–adrenal (HPA) axis, producing hypercortisolemia, adrenergic overdrive, oxidative stress, and systemic inflammation, which sustain blood pressure elevation [31,32,33]. Neuroimaging studies revealed stress-induced remodeling of the hippocampus, amygdala, and prefrontal cortex, impairing stress reactivity and cardiovascular control [34,35]. 

In addition to these general mechanisms, continuous activation of the HPA axis and sympathetic nervous system promotes the release of pro-inflammatory cytokines such as interleukin-6 (IL-6), tumor necrosis factor-alpha (TNF-α), and interleukin-1 beta (IL-1β) while enhancing oxidative stress through increased levels of reactive oxygen species (ROS) and malondialdehyde (MDA) and reduced antioxidant defenses such as glutathione (GSH). These processes contribute to vascular remodeling, endothelial dysfunction, and arterial stiffness, forming the biological substrate that links chronic psychological stress to sustained hypertension [14].

Telomeres serve as biomarkers of psychological stress that can lead to disease. Through psycho-neuro-endocrine connections, molecular mechanisms linking stress, telomeres, and diseases involve stress hormones, reactive oxidative species (ROS), and inflammation [36,37]. Chronic psychological stress accelerates telomere shortening by increasing oxidative damage and dysregulating the HPA axis, promoting premature vascular aging and elevated hypertension risk. As a result, telomere attrition functions as a biological link between psychosocial stress and cardiovascular dysfunction.

Many cross-sectional studies show a positive bidirectional association between anxiety and hypertension, and anxiety can be a significant predictor of the evolution of heart disease [38,39]. 

Both genetic and epigenetic factors are involved in determining anxiety, hypertension, and vulnerability to stress, and immunogenic personality traits play an important role. Although genetic and epigenetic mechanisms are known to modulate stress and blood pressure reactivity, these were beyond the scope of the present study and are mentioned only to contextualize psychological vulnerability within a broader biopsychosocial framework. Of these traits, self-efficacy should be considered a protective factor against stress. High self-efficacy acts as a buffer factor, causing a lower vulnerability to stress, anxiety, and depression, and motivates hypertensive patients to develop sanogenic behavior (adequate nutrition, exercise, giving up risky behaviors) and treatment adherence [40,41,42,43,44,45,46].

Reviews confirmed that these mechanisms act as long-term drivers of HTN and its complications [47,48,49].

### 1.5. Gender Differences and the Impact of Menopause

Gender plays a significant role in the epidemiology of HTN. Men tend to develop HTN earlier in life, whereas women experience a steep rise in prevalence after menopause, largely due to the loss of estrogen-mediated vascular protection [50,51,52,53]. In addition to biological mechanisms, the menopausal transition is frequently associated with heightened stress sensitivity, anxiety, and sleep disturbances, which may further contribute to blood pressure elevation [54,55,56]. 

These observations underscore the need for gender-sensitive approaches to hypertension management, particularly in middle-aged and older women.

### 1.6. Self-Efficacy as a Protective Factor

Psychological resilience traits, particularly self-efficacy, play a critical role in moderating the effects of stress on blood pressure. Bandura defined self-efficacy as the belief in one’s ability to mobilize cognitive and motivational resources to achieve desired outcomes, and it is increasingly recognized as a protective factor in hypertension (HTN) [57,58,59,60]. Higher levels of self-efficacy are associated with better treatment adherence, greater risk awareness, and healthier lifestyle behaviors [41,42]. 

Conversely, low self-efficacy is linked to maladaptive coping, poor compliance, anxiety, and elevated cardiovascular risk [61,62].

### 1.7. Integrating Psychological and Digital Interventions

Recent interventions highlight the benefits of combining psychological and technological approaches in hypertension care. Mindfulness-based interventions have been shown to reduce blood pressure and improve emotional outcomes [63,64,65].

Mobile health (mHealth) tools have been shown to improve adherence and lower blood pressure by clinically meaningful margins [66,67]. 

Telemonitoring with professional case management produces superior long-term blood pressure reductions compared with telemonitoring alone [68,69,70]. 

Collectively, these findings support the integration of psychosocial and digital interventions into cardiovascular management.

### 1.8. Rationale and Study Aim

Although the association between psychological distress and hypertension has been widely explored in international literature, data from Eastern Europe—and particularly from Romania—remain scarce. Most existing studies have been conducted in Western populations and focus on either stress or anxiety in isolation, seldom incorporating multiple psychosocial variables such as perceived stress, self-efficacy, and personality traits within the same analytic framework [71]. Furthermore, little is known about how these psychological dimensions interact to influence blood pressure regulation and adherence to antihypertensive therapy in culturally and socioeconomically diverse settings [72].

Romania presents a particularly relevant context for this investigation. The country faces a high prevalence of hypertension and a rising burden of stress-related disorders, but integrated behavioral–cardiovascular research is limited. Understanding the relationship between psychological vulnerability and cardiovascular health in this population is essential for designing integrated, cost-effective preventive strategies.

The present study addresses this gap by examining the relationships between stress, anxiety, self-efficacy, and Type A behavior in Romanian adults with and without hypertension. Its goal is to identify modifiable psychological factors that may contribute to cardiovascular risk and to accentuate personalized, non-pharmacological interventions that could complement standard medical treatment.

Based on the literature and preliminary observations, the study formulated the following hypotheses:

1. Hypertensive adults will exhibit higher levels of perceived stress and anxiety than normotensive controls.
2. Hypertensive adults will demonstrate lower self-efficacy, reflecting reduced perceived control over stress and health behaviors.
3. Type A personality traits will show no significant independent association with hypertension when analyzed alongside other psychological factors.
4. These associations will remain significant after adjusting for demographic variables, including age, sex, and occupation.

By testing these hypotheses, the study aims to provide region-specific evidence on the psychological factors associated with hypertension and to support the integration of mental health screening and behavioral interventions into routine cardiovascular care in Romania.

## 2. Materials and Methods

### 2.1. Study Design and Participants

This research was designed as a retrospective, case–control study conducted between February and June 2024. A total of 215 adults participated, of whom 104 were diagnosed with arterial hypertension (case group) and 111 without hypertension (control group).

Inclusion criteria: age ≥ 18 years; confirmed diagnosis of arterial hypertension according to ESC/ESH 2018 guidelines (≥140/90 mmHg or ongoing antihypertensive treatment); informed consent to participate.

Blood pressure values were measured at enrollment using standardized office procedures, in accordance with the 2018 ESC/ESH guidelines. Each participant underwent two seated measurements, taken one to two minutes apart, using a validated automatic sphygmomanometer; the mean value was recorded. For participants under antihypertensive treatment, classification as ‘hypertensive’ required either current medication use or documented prior diagnosis with persistent readings ≥140/90 mmHg despite therapy.

Exclusion criteria: age < 18 years, presence of psychiatric disorders diagnosed prior to enrollment, cognitive impairment, or refusal to consent.

The sample size was constrained by feasibility and recruitment limitations. Nevertheless, a post hoc power analysis (G*Power 3.1) indicated that, to detect a medium effect size (Cohen’s d = 0.5) with α = 0.05 and power = 0.80, a minimum of 102 participants per group was required. Our study thus achieved sufficient statistical power to test the main hypotheses.

Although the achieved sample size met the requirements of the post hoc power analysis for detecting medium effect sizes, it does not fully guarantee representativeness of the broader Romanian hypertensive population. The study was conducted on a volunteer sample recruited from the Family Doctor’s office and via an online survey (Google Forms^®^). Therefore, the findings should be interpreted primarily as hypothesis-generating and illustrative of psycho-emotional patterns, rather than as definitive prevalence estimates, and can be considered representative of a sample.

### 2.2. Recruitment and Ethical Considerations

Participants were recruited through two complementary strategies:

- An online survey (Google Forms®) designed for individuals with mobility difficulties who could not attend in person, distributed via mailing lists of healthcare professionals and academic communities, social media platforms (Facebook and LinkedIn groups related to health, psychology, and lifestyle), and professional messaging applications such as WhatsApp;
- Direct enrollment through family medicine offices for the remaining participants.

Each participant received detailed study information and provided informed consent electronically (online) or in writing (in the office) prior to participation. This dual recruitment strategy was deliberately chosen to ensure access to a geographically diverse adult population across Romania, encompassing both urban and rural areas, while also accommodating participants with limited mobility. 

A total of 92 participants (42.8%) were recruited online, and 123 (57.2%) were recruited through family medicine offices. The two subsamples did not differ significantly in age, gender, or educational level (all *p* > 0.05). However, a higher proportion of urban residents participated online, while rural participants were mainly recruited via family physicians. Psychological scores did not differ significantly between recruitment modes, suggesting minimal bias was introduced by the data collection format. Detailed comparisons are available in Supplementary Table S1.

Online surveys have been increasingly validated in epidemiological and psychological research for their efficiency, cost-effectiveness, and ability to capture large, heterogeneous samples. Complementary recruitment through family medicine offices provided additional representativeness by including patients encountered in routine clinical practice.

The study was conducted in accordance with the Declaration of Helsinki and was approved by the Ethics Committee of University of Medicine and Pharmacy “Carol Davila”, Bucharest, Romania (protocol code no. 532/10.01.2024; approval date: 1 October 2024). Confidentiality of all data was strictly maintained.

Nonetheless, we acknowledge that online recruitment may have introduced selection bias, as digitally literate and health-conscious individuals are more likely to participate. To mitigate this risk, the study invitation emphasized inclusivity and was disseminated across platforms with a broad demographic reach. No incentives were provided, thereby reducing the likelihood of participation driven by secondary gain. The implications of this recruitment strategy for generalizability are further addressed in the Strengths and Limitations Section.

### 2.3. Psychometric Instruments

Validated psychological instruments widely used in clinical and epidemiological settings were applied. Each test was selected for its relevance to stress, anxiety, and self-efficacy in the context of cardiovascular disease. All instruments were administered in Romanian using previously validated versions that demonstrated adequate psychometric properties in local samples [73,74,75,76,77,78,79,80,81] and applied in a self-administered format.

- Stress Vulnerability Scale (SVS): This instrument includes 20 items, each rated on a five-point Likert scale (1 = never to 5 = always), assessing susceptibility to stress-related maladaptive responses. Higher scores indicate greater vulnerability to stress. In the present sample, internal consistency was Cronbach’s α = 0.87, consistent with previously reported values (α = 0.82) [73].
- Perceived Stress Scale (PSS-10): A 10-item measure assessing subjective stress over the previous month, with responses rated on a five-point Likert scale (0 = never to 4 = very often). Four items are reverse-coded. Higher scores reflect higher perceived stress. In the current sample, reliability was α = 0.84, within the typical range (0.78–0.91) [74].
- State–Trait Anxiety Inventory (STAI-1 and STAI-2): The instrument comprises **40 items**, divided equally between the State Anxiety (STAI-S) and Trait Anxiety (STAI-T) subscales (each 20 items). Items are scored on a four-point Likert scale (1 = not at all to 4 = very much so), with higher scores indicating greater anxiety. In this study, internal consistency was α = 0.91 for STAI-S and α = 0.89 for STAI-T, comparable with published data (α > 0.90) [75].
- General Self-Efficacy Scale (GSES): Contains 10 items rated on a four-point Likert scale (1 = not at all true to 4 = exactly true). Higher scores indicate stronger perceived self-efficacy in coping with daily challenges. Reliability in the present sample was **α = 0.88**, consistent with prior research (α = 0.86–0.94) [76].
- Jenkins Activity Survey (JAS): Used to assess the Type A behavior pattern, this questionnaire includes 52 items, of which 21 items form the Type A behavior index, scored on a six-point Likert scale. Higher scores denote a stronger Type A behavioral tendency. Internal consistency for the total scale in this sample was α = 0.82, consistent with earlier studies (α ≈ 0.77) [77].

Psychometric characteristics of all instruments used in the study are summarized in Table 1. The Cronbach’s α coefficients correspond to the present study, while the reference values are drawn from the original international validation studies.

To minimize potential order effects, all instruments were presented in the same standardized sequence: SVS, PSS-10, STAI (Forms 1 and 2), GSES, and JAS. This order was chosen to progress from general stress measures to more specific psychological constructs, reducing response fatigue and ensuring consistency across participants.

### 2.4. Baseline Characteristics

Demographic and socioeconomic data collected included age (years, stratified by decades), gender (male/female), place of residence (urban/rural), occupational profile (mental overload, physical overload, negative emotional exposure, or other), educational level and marital status.

This ensured a baseline comparison between groups beyond psychometric outcomes.

### 2.5. Statistical Analysis

An a priori power analysis was conducted using GPower (version 3.1.9.7, Heinrich Heine University, Düsseldorf, Germany) to determine the minimum sample size required for detecting medium effect sizes (Cohen’s d = 0.5) at a power level of 0.80 and a two-tailed α = 0.05. This analysis indicated a minimum of 176 participants; the final sample of 215 thus provided adequate statistical power for the primary comparisons between hypertensive and normotensive groups.

Data were analyzed using IBM SPSS Statistics 28.0 (IBM Corp., Armonk, NY, USA). Prior to analysis, assumptions of normality and homogeneity of variance were verified using the Shapiro–Wilk test and Levene’s test, respectively.

- For normally distributed continuous variables, independent-samples t-tests were applied.
- For non-normal distributions, the Mann–Whitney U test was considered as a sensitivity analysis.

All continuous variables were first tested for normality using the Shapiro–Wilk test. Most psychometric measures followed an approximately normal distribution; however, the Stress Vulnerability Scale (SVS) and State Anxiety (STAI-S) scores showed slight deviations from normality. Therefore, Mann–Whitney U tests were additionally performed as sensitivity analyses for these variables. As results were consistent with those obtained using parametric tests, only *t*-test outcomes are reported in the main tables for clarity.

- Categorical variables were analyzed using chi-square or Fisher’s exact test, as appropriate.
- Effect sizes were reported as Cohen’s d (for mean differences) and Cramer’s V (for categorical associations).
- A two-tailed p-value <0.05 was considered statistically significant.

**Figure 1:**
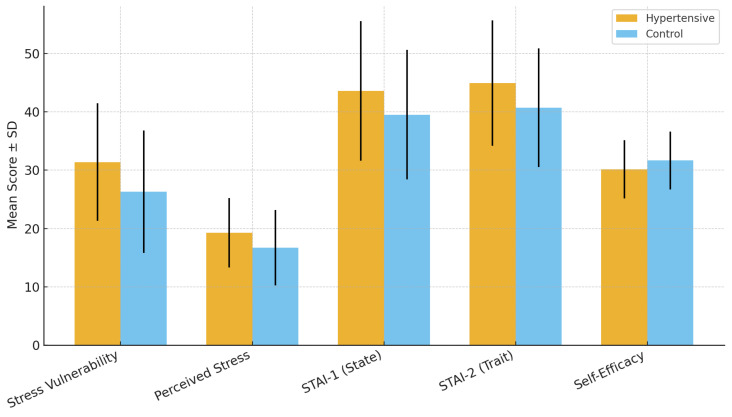
Comparison of mean stress vulnerability (SVS) and perceived stress (PSS-10) scores (±SD) between hypertensive and control participants. Hypertensive patients reported significantly higher levels of both stress vulnerability and perceived stress. Error bars represent standard deviations.

**Figure 2:**
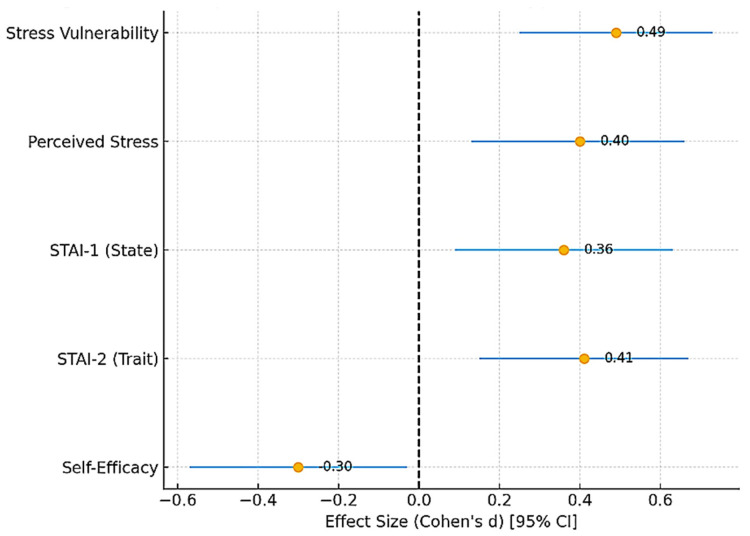
Comparison of mean state anxiety (STAI-1) and trait anxiety (STAI-2) scores (±SD) between hypertensive and control participants. Hypertensive patients demonstrated significantly higher state and trait anxiety, with moderate effect sizes. Error bars represent standard deviations.

**Figure 3:**
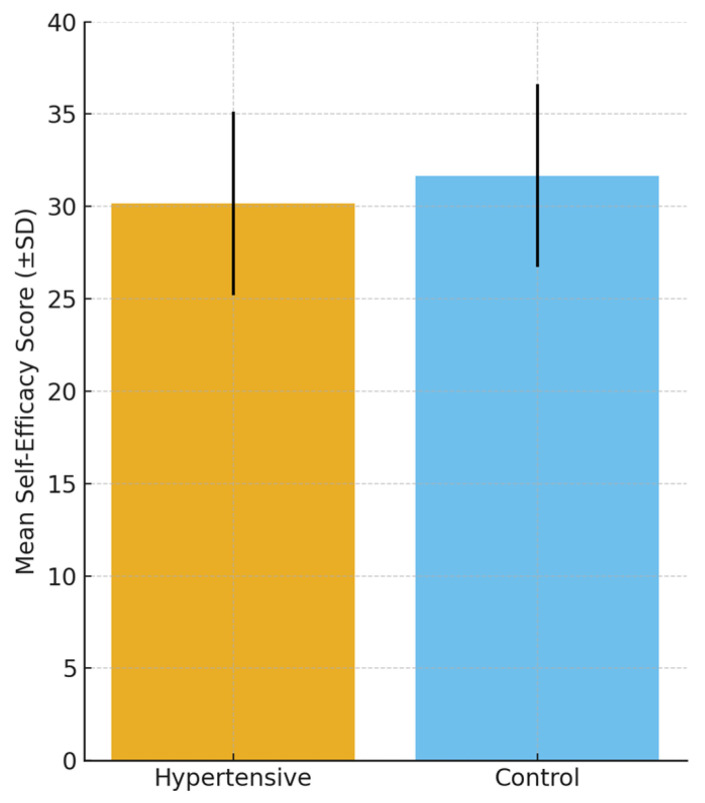
Comparison of mean self-efficacy scores (General Self-Efficacy Scale, GSES; ±SD) between hypertensive and control participants. Self-efficacy was significantly lower in hypertensive patients. Error bars represent standard deviations.

**Figure 4:**
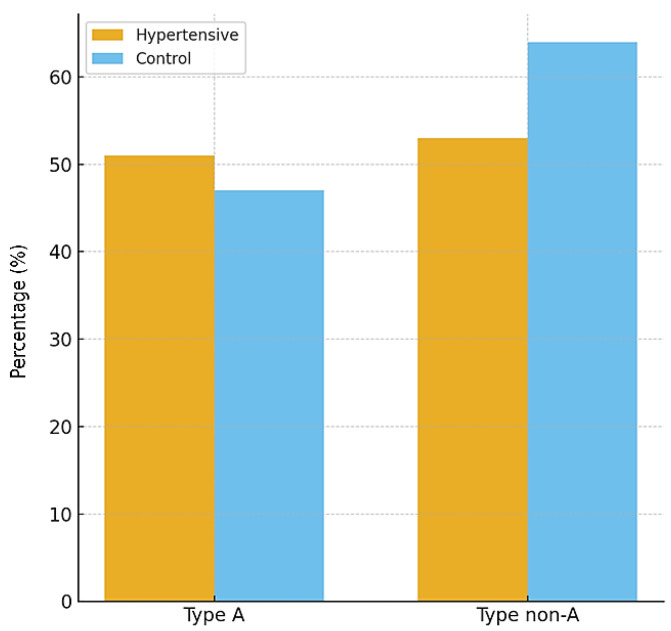
Distribution of psychobehavioral Type A patterns between hypertensive and control participants. No statistically significant differences were observed between groups.

**Figure 5:**
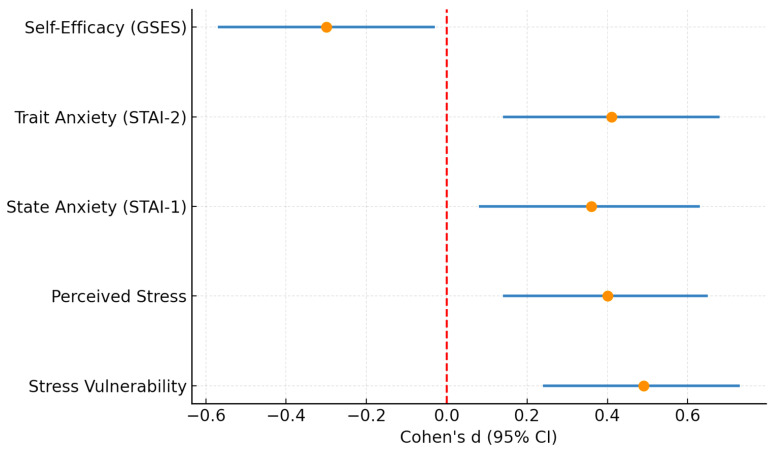
Forest plot summarizing standardized effect sizes (Cohen’s d) with 95% confidence intervals for all psychometric outcomes in hypertensive versus control participants. Positive values indicate higher scores in the hypertensive group, while negative values indicate higher scores in the control group. Stress vulnerability, perceived stress, and both state and trait anxiety showed moderate positive effect sizes, whereas self-efficacy displayed a small-to-moderate negative effect size. The dashed red line at 0 represents the null effect (no difference between groups).

To ensure transparency and reproducibility, descriptive data are presented as mean ± standard deviation (SD) for continuous variables and as absolute counts and percentages for categorical variables. To further enhance transparency and reproducibility, effect sizes (Cohen’s d, Cramer’s V) were complemented with 95% confidence intervals, which are presented in Figure 1, Figure 2, Figure 3, Figure 4 and Figure 5 and Table 2, Table 3, Table 4, Table 5 and Table 6.

In addition, exploratory multivariable analyses were performed to evaluate potential confounding by age, gender, and occupational profile, given the observed differences between groups. These covariates were entered as control variables in multiple linear regression models with each psychometric score as the dependent variable. The inclusion of these covariates did not substantially alter the significance or direction of the main group effects, indicating that the associations between hypertension status and psychological outcomes were largely independent of demographic factors.

## 3. Results

### 3.1. Baseline Characteristics

A total of 215 participants were analyzed: 104 in the hypertensive group and 111 in the control group. Demographic and socioeconomic characteristics are summarized in Table 2.

**Table 2:** Baseline demographic and socioeconomic characteristics of participants.

Variable	Hypertensive (*n* = 104)	Control (*n* = 111)	*p*-Value
**Age (years), mean ± SD**	54.2 ± 9.6	52.7 ± 8.9	0.24
**Age groups, *n* (%)**			
18–39	4 (4%)	13 (12%)	–
40–49	27 (26%)	36 (32%)	–
50–59	46 (44%)	50 (45%)	–
60–69	23 (22%)	11 (10%)	–
**Gender, *n* (%)**			
Female	78 (75%)	95 (86%)	0.06
Male	26 (25%)	16 (14%)	–
**Residence, *n* (%)**			
Urban	86 (83%)	94 (85%)	0.72
Rural	18 (17%)	17 (15%)	–
**Occupation, *n* (%)**			
Mental overload	38 (37%)	64 (58%)	<0.01
Emotional stress	29 (28%)	22 (20%)	–
Physical overload	19 (18%)	10 (9%)	–
Other	18 (17%)	15 (13%)	–

The mean age was comparable between groups (54.2 ± 9.6 years in hypertensive patients vs. 52.7 ± 8.9 years in controls, *p* = 0.24). When stratified by age groups, the majority of participants fell within the 40–59-year range in both groups, with no significant between-group differences.

The gender distribution revealed a predominance of females in both groups (75% in the hypertensive group vs. 86% in the control group, *p* = 0.06), although the difference did not reach statistical significance. The higher proportion of women, especially among hypertensive participants, may partly reflect the age-related increase in hypertension risk observed in postmenopausal women. However, since menopausal status was not specifically recorded in this study, this interpretation should be considered exploratory.

The majority of participants resided in urban areas (83% vs. 85%, *p* = 0.72). Regarding occupational profile, hypertensive participants were less frequently employed in mentally demanding occupations compared to controls (37% vs. 58%, *p* < 0.01). Other occupational categories (emotional stress, physical overload, or other) showed no significant differences. This occupational difference was further examined in multivariable analyses controlling for age and gender, and it did not substantially alter the associations between hypertension status and psychological outcomes. Detailed recruitment-related comparisons (online vs. office-based participants) are provided in Supplementary Table S1.

### 3.2. Stress Vulnerability and Perceived Stress

Hypertensive participants reported significantly higher stress vulnerability and perceived stress levels compared with controls. The mean SVS score was 31.39 ± 10.09 in the hypertensive group versus 26.29 ± 10.49 in controls (*p* < 0.001, Cohen’s d = 0.49), while the mean PSS-10 score was 19.27 ± 5.97 versus 16.72 ± 6.44, respectively (*p* = 0.003, Cohen’s d = 0.40). These results indicate moderate effect sizes, suggesting that stress-related dimensions play a measurable role in hypertensive status.

Table 3 summarizes the results, while Figure 1 graphically illustrates the group differences, showing consistently higher stress vulnerability and perceived stress among hypertensive participants. 

**Table 3:** Stress vulnerability and perceived stress scores in hypertensive and control groups.

Scale	Hypertensive (*n* = 104) Mean ± SD	Control (*n* = 111) Mean ± SD	Mean Difference (95% CI)	*p*-Value	Cohen’s d (95% CI)
Stress Vulnerability Scale (SVS)	31.39 ± 10.09	26.29 ± 10.49	+5.11 (2.30–7.92)	<0.001	0.49 (0.24–0.73)
Perceived Stress Scale (PSS-10)	19.27 ± 5.97	16.72 ± 6.44	+2.55 (0.90–4.21)	0.003	0.40 (0.14–0.65)

To complement the tabular data, Figure 1 illustrates the mean scores with standard deviations for both groups, showing consistently higher stress measures in hypertensive participants.

### 3.3. Anxiety (State and Trait)

Both state anxiety (STAI-1) and trait anxiety (STAI-2) scores were significantly higher in the hypertensive group compared to controls. Hypertensive participants reported a mean STAI-1 score of 43.58 ± 11.95 versus 39.50 ± 11.08 in the control group (*p* = 0.010, Cohen’s d = 0.36). Similarly, mean STAI-2 scores were 44.91 ± 10.74 versus 40.68 ± 10.16, respectively (*p* = 0.003, Cohen’s d = 0.41). These findings confirm the strong association between hypertension and heightened anxiety.

Detailed scores are presented in Table 4, while Figure 2 illustrates these results visually, highlighting the higher mean levels of both state and trait anxiety in hypertensive participants.

**Table 4:** Anxiety scores in hypertensive and control groups.

Scale	Hypertensive (*n* = 104) Mean ± SD	Control (*n* = 111) Mean ± SD	Mean Difference (95% CI)	*p*-Value	Cohen’s d (95% CI)
STAI-1 (State Anxiety)	43.58 ± 11.95	39.50 ± 11.08	+4.07 (0.96–7.18)	0.010	0.36 (0.08–0.63)
STAI-2 (Trait Anxiety)	44.91 ± 10.74	40.68 ± 10.16	+4.24 (1.48–7.01)	0.003	0.41 (0.14–0.68)

These differences are further illustrated in Figure 2, where hypertensive participants show consistently higher mean anxiety scores than controls.

### 3.4. Self-Efficacy

Self-efficacy levels, as measured by the General Self-Efficacy Scale (GSES), were significantly lower among hypertensive participants compared to controls. The mean score in the hypertensive group was 30.16 ± 4.98 versus 31.67 ± 4.95 in controls (*p* = 0.028, Cohen’s d = 0.30). Although the effect size was small to moderate, this finding suggests that reduced self-efficacy may represent a psychological vulnerability factor in hypertension.

Results are summarized in Table 5, and Figure 3 provides a graphical comparison, emphasizing the lower self-efficacy scores among hypertensive patients. The group differences are also visualized in Figure 3, which highlights the lower mean self-efficacy score among hypertensive patients compared with controls. Whether this reduced self-efficacy represents a predisposing trait contributing to hypertension onset or a psychological consequence of living with a chronic condition remains an open question and is further explored in the Discussion Section.

**Table 5:** Self-efficacy scores in hypertensive and control groups.

Scale	Hypertensive (*n* = 104) Mean ± SD	Control (*n* = 111) Mean ± SD	Mean Difference (95% CI)	*p*-Value	Cohen’s d (95% CI)
General Self-Efficacy Scale (GSES)	30.16 ± 4.98	31.67 ± 4.95	−1.50 (−2.84 to −0.16)	0.028	0.30 (0.03–0.57)

### 3.5. Psychobehavioral Type A

The distribution of psychobehavioral Type A did not differ significantly between hypertensive and control participants. In the hypertensive group, 49% exhibited a Type A behavior pattern compared with 42% in the control group (*p* = 0.33). This result suggests that, within the studied population, Type A behavior was not independently associated with hypertension.

Table 6 presents the distribution across groups, and Figure 4 illustrates the proportions, confirming the absence of statistically significant differences. 

**Table 6:** Distribution of psychobehavioral Type A in hypertensive and control groups.

Variable	Hypertensive (*n* = 104)	Control (*n* = 111)	*p*-Value
Type A, *n* (%)	51 (49%)	47 (42%)	0.33
Type non-A, *n* (%)	53 (51%)	64 (58%)	–

The proportions are illustrated in Figure 5, confirming the absence of statistically significant differences between the two groups.

### 3.6. Summary of Findings

In summary, hypertensive patients showed significantly greater stress vulnerability, perceived stress, and anxiety compared with controls, alongside lower self-efficacy. No significant differences were observed in psychobehavioral Type A between groups.

To provide an integrated overview of these findings, Figure 5 presents a forest plot summarizing the standardized effect sizes (Cohen’s d) with 95% confidence intervals for all psychometric outcomes. This synthesis underlines consistent moderate positive effects for stress and anxiety measures and a small-to-moderate negative effect for self-efficacy.

## 4. Discussion

The present study examined the psychological coordinates in arterial hypertension in a Romanian adult population, with a focus on stress vulnerability, perceived stress, anxiety, self-efficacy, and Type A psychobehavioral patterns. Hypertensive participants exhibited significantly higher perceived stress and anxiety and lower self-efficacy compared with normotensive controls, whereas no differences were found in Type A behavior. These results confirm our hypotheses and align with previous findings indicating that psychosocial stress, maladaptive emotional regulation, and diminished self-efficacy are central mechanisms linking psychological functioning with hypertension onset and control [19].

Chronic stress exposure activates neuroendocrine pathways—particularly the hypothalamic–pituitary–adrenal (HPA) axis and sympathetic nervous system—promoting high cortisol levels, catecholamine release, endothelial dysfunction, and sustained blood pressure elevation [82]. The higher stress vulnerability observed in hypertensive individuals may therefore reflect both heightened physiological reactivity and impaired stress coping [83]. On the contrary, reduced self-efficacy can exacerbate this cycle by limiting confidence in personal ability to adhere to antihypertensive treatment, maintain healthy lifestyle behaviors, and effectively regulate stress responses [20].

The absence of significant differences in Type A behavioral traits suggests that personality typologies may have a lesser impact on blood pressure regulation than do dynamic psychological processes, such as perceived stress and anxiety [84,85]. This finding is consistent with contemporary evidence showing that traditional Type A behavior models—once considered central to cardiovascular risk—are now viewed as less predictive than emotional and cognitive factors influencing health behaviors [86].

All together, these results underline the multifactorial nature of hypertension and the need for integrative models that include psychological assessment in cardiovascular management, especially in Eastern Europe, where hypertension prevalence and stress exposure remain excessively high [87,88].

### 4.1. Contribution to Current Evidence

Our results provide regional evidence that hypertension is consistently accompanied by adverse psychological characteristics. Recent longitudinal research has revealed mixed findings regarding anxiety and HTN. For example, Jones et al. (2025) found that anxiety was associated with self-reported but not clinically measured systolic blood pressure, emphasizing that association may depend on diagnostic modality and perception rather than objective criteria [88]. Conversely, systematic reviews confirm that depression and anxiety often precede or accompany HTN and worsen its outcomes [89,90,91,92].

By documenting significant differences in stress, anxiety, and self-efficacy between hypertensive and control groups, our study adds local support for integrating psychological assessment into hypertension management. It emphasizes that the challenge is not only cardiovascular but also psycho-emotional, mirroring observations across diverse cultural and socioeconomic contexts [93,94,95]. Importantly, the integrated analysis summarized in Figure 5 shows that these associations are consistent across multiple psychometric domains, with moderate effect sizes for stress and anxiety and a small-to-moderate negative effect for self-efficacy, reinforcing their clinical relevance.

### 4.2. Stress and Stress Vulnerability

Hypertensive participants in our study showed greater stress vulnerability and perceived stress, with effect sizes in the moderate range. These results are in line with prior work demonstrating that stress contributes to blood pressure elevation via both behavioral and physiological mechanisms [20,21].

Context matters: Denche-Zamorano et al. (2023) showed that Spanish hypertensive patients with higher stress and anxiety engaged in less physical activity, compounding cardiovascular risk [96]. Boukhari et al. (2024) reported that over half of Moroccan hypertensives experienced depression strongly linked to stress and social factors [23]. In Afghanistan, Hamrah et al. (2018) found high distress among hypertensive patients, shaped by conflict and limited healthcare access [22]. These findings underscore that stress is a universal risk factor, but its manifestation is highly influenced by environment and resources.

Recent interventional research adds another perspective. A 2024 randomized trial from China found that an eight-week mindfulness program reduced systolic blood pressure by ~12 mmHg, lowered stress, and improved self-efficacy in hypertensive patients with comorbid depression and anxiety [97]. Such evidence indicates that stress is not merely associated with HTN but represents a modifiable therapeutic target, strengthening the need to integrate stress management strategies into HTN care.

### 4.3. Anxiety and Hypertension

Both state and trait anxiety were higher among hypertensive participants in our study. This is in line with global evidence of a bidirectional relationship between anxiety and HTN. For example, Hamrah et al. [22] and Thomas et al. [75] reported elevated anxiety prevalence in hypertensive populations. At the same time, a systematic review in young U.S. adults confirmed bidirectional associations between anxiety/depression and hypertension, particularly among women and low-income groups [91].

Prognostic implications are equally important. Huang et al. (2024) demonstrated that hypertensive patients with comorbid depression had significantly higher all-cause and cardiovascular mortality [25]. Similarly, a recent meta-analysis confirmed that depression increases the risk of developing HTN by more than 50% [24,26]. Although some studies note partial attenuation after adjusting for health behaviors such as smoking or medication adherence, the persistence of these associations across large datasets suggests that anxiety may exert an independent effect [98].

Taken together, these data and our findings reinforce that anxiety should not be regarded as a secondary concern but as a clinically meaningful factor in hypertension care. Routine screening for anxiety—even in patients without formal psychiatric diagnoses—may improve risk stratification and guide supportive interventions.

### 4.4. Gender Differences and the Role of Menopause

An important finding in our study is the predominance of women in both groups, particularly among hypertensive participants. This distribution is consistent with epidemiological evidence showing that the prevalence of HTN increases substantially in women after menopause, when the loss of estrogen protection may contribute to vascular dysfunction, heightened sympathetic activity, and adverse metabolic changes such as insulin resistance and dyslipidemia [99].

Beyond these biological mechanisms, the menopausal transition is frequently accompanied by increased stress sensitivity, anxiety, and sleep disturbances, which may further elevate blood pressure and impair treatment adherence [100]. Previous studies also indicate that postmenopausal women are more vulnerable to psychosocial stressors and display higher cardiovascular risk compared with men of the same age [101,102].

Although menopausal status was not directly assessed in this study, the predominance of women among hypertensive participants may partly reflect age-related hormonal changes and their interaction with psycho-emotional vulnerability.

These findings support the importance of implementing gender-sensitive approaches in the psychosocial assessment and management of HTN.

### 4.5. Self-Efficacy as a Protective Factor

A key contribution of our study is the identification of lower self-efficacy in participants with HTN. While effect sizes were smaller than for stress or anxiety, the clinical importance is considerable, since self-efficacy is modifiable.

Recent studies underscore this. Soylu and Tanrıverdi (2024) found that self-efficacy predicted adherence to antihypertensive treatment [103]. Lim (2024) reported that the use of digital patient portals improved self-efficacy and blood pressure management [104]. Salmanpour et al. (2025) demonstrated that self-efficacy and health literacy independently predicted quality of life in hypertensive populations [46].

Our results echo these findings, suggesting that low self-efficacy may hinder coping and adherence among Romanian hypertensives. Significantly, self-efficacy can be strengthened through educational programs, motivational counseling, and digital interventions. Self-efficacy represents a dynamic and modifiable clinical target with direct potential to improve both psychological well-being and hypertension outcomes.

### 4.6. Psychobehavioral Type A

Contrary to early psychosomatic theories, we found no significant differences in type A behavior between hypertensive and control groups. This is consistent with recent personality findings indicating that global type A is no longer an independent predictor of cardiovascular risk once factors such as stress reactivity and hostility are accounted for [77]. For instance, a 2025 Japanese cohort study using the Big Five framework reported that conscientiousness was protective, while openness sometimes correlated with higher risk, but type A per se was not predictive [105].

Our results, therefore, reflect a broader scientific shift: the clinical focus should move from rigid typologies such as type A to modifiable psychosocial factors—stress, anxiety, and self-efficacy—that show stronger and more consistent associations with hypertension.

### 4.7. Interventions and Translation to Practice

The psycho-emotional differences identified in our study—elevated stress and anxiety combined with lower self-efficacy—directly correspond to targets of interventions that have already demonstrated efficacy in hypertension management.

Stress and anxiety reduction: Mindfulness-Based Blood Pressure Reduction (MB-BP) programs have been shown to improve interoceptive awareness, emotional regulation, and blood pressure control, with a concomitant reduction in anxiety and depressive symptoms [63,64,102]. These approaches are particularly relevant given the heightened stress reactivity observed among hypertensive participants in our cohort. Complementary techniques, such as cognitive–behavioral therapy (CBT), can help patients by identifying maladaptive patterns, improving coping strategies, and reducing physiological stress responses.

Enhancing self-efficacy and treatment adherence: The lower self-efficacy scores identified among hypertensive individuals in our study indicate the need for structured motivational and educational interventions. Motivational interviewing and personalized self-management training have been shown to increase patients’ confidence in managing their illness and adhering to lifestyle changes and medication regimens. In the Romanian healthcare context, these interventions could feasibly be implemented through brief sessions in family medicine offices, supported by psychologist group programs.

Digital and telehealth interventions: Digital platforms (mHealth tools, mobile applications, and telemonitoring systems) are accessible and cost-effective options to strengthen psychological support and adherence. Meta-analyses confirm that these strategies reduce systolic and diastolic blood pressure while improving self-efficacy and behavioral consistency [67,106]. Telehealth systems have proven scalable in diverse clinical settings [107]; for Romania, integrating nurse-led or psychologist-assisted telemonitoring may help reach rural and underserved populations.

Taken together, these approaches align closely with the psycho-emotional profile observed in our study—addressing elevated stress and anxiety while fostering self-efficacy as a protective psychological resource. Therefore, future research in Romania and Eastern Europe should prioritize the cultural adaptation and evaluation of mindfulness, CBT, and mHealth-based programs to ensure their effective integration into cardiovascular care pathways.

### 4.8. Clinical and Public Health Implications

Our findings highlight that HTN cannot be addressed effectively without acknowledging its psychological dimensions. Hypertensive patients in our study reported a dual burden: elevated stress and anxiety combined with lower self-efficacy. These characteristics may act synergistically, exacerbating poor adherence and worsening cardiovascular outcomes.

At the clinical level, psychosocial screening should be incorporated into routine hypertension care. Validated, short instruments such as the PSS-10, STAI, and GSES are feasible in primary care and cardiology settings and can help identify at-risk patients early. Evidence shows that even brief interventions, such as stress management, motivational interviewing, or mindfulness practices, can reduce both psychological distress and blood pressure [63,64,65]. Digital platforms, including patient portals and mHealth apps, have also demonstrated efficacy in reinforcing adherence and self-management [67].

At the public health level, policies should move towards integrated care models that bring together physicians, psychologists, and nurses. Scaling up digital health and telemonitoring strategies is particularly important in Eastern Europe, where the control of HTN remains suboptimal [1,4,108,109,110]. By including psychosocial support into cardiovascular care pathways, health systems can better address disparities and improve outcomes. Our findings—synthesized in Figure 5—reinforce that stress, anxiety, and self-efficacy are central, modifiable targets for both clinical practice and public health interventions.

### 4.9. Conceptual Model: Linking Hypertension, Stress, Anxiety, and Self-Efficacy

Research findings indicate that hypertension develops through an integrated biological–psychological system, which links stress perception to anxiety and self-efficacy [111]. The hypothalamic–pituitary–adrenal (HPA) axis and the sympathetic nervous system (SNS) are activated by prolonged psychological stress, leading to elevated cortisol, adrenaline, and noradrenaline levels [112]. Neuroendocrine changes in the body result in damaged endothelial tissue, elevated blood vessel resistance, and minimal inflammation, which help sustain elevated blood pressure [113].

The psychological aspects of stress and anxiety influence how people process information, manage their emotions, and handle difficult situations [114]. People who lack self-efficacy tend to use avoidance and emotional coping methods, which intensify their physical responses and prevent them from following health-promoting behaviors, including medication use, exercise, and dietary management [115]. People with strong self-efficacy develop better resilience, which helps them handle stress through effective coping strategies and better behavioral control [116].

These pathways exist in a state of ongoing interaction. The combination of stress and anxiety causes blood pressure to rise through sympathetic nervous system overactivity, but people with weak self-efficacy experience worse blood pressure increases because their coping abilities are reduced [117,118]. The ongoing process creates a psychobiological feedback system that links emotional control problems to the development of heart disease.

The proposed relationship exists as a smooth spectrum rather than distinct categories because psychosocial stress and low self-efficacy affect blood pressure levels from prehypertension to established hypertension, with more pronounced clinical symptoms at higher systolic and diastolic pressure values [119]. The strength of these relationships depends on individual genetic makeup, how people react to stress, and their total exposure to life events [120].

The conceptual diagram in Supplementary Figure S1 presents a visual representation of psychological distress, coping mechanisms, neuroendocrine responses, and cardiovascular outcomes, with self-efficacy as a connecting factor between biological and behavioral elements.

### 4.10. Practical Implications for Clinical and Public Health Settings

Pragmatic alignment with ESC 2025. In the 2025 ESC Clinical Consensus Statement on mental health and cardiovascular disease, treatment decisions are placed in an absolute-risk framework and measured against clinically relevant outcomes [5]. For adults with elevated BP or hypertension, implementation is essential: prioritize validated out-of-office BP, follow 120–129/70–79 mmHg on-treatment when tolerated, and use simple, scalable strategies that improve adherence. Within this paradigm, the psycho-emotional profile observed here—higher stress and anxiety with lower self-efficacy—identifies modifiable levers to support risk reduction and BP control.

Operational steps (primary care and cardiology) include (i) brief psychosocial screening (PSS-10, STAI, GSES) integrated into BP visits; (ii) structured counseling (CBT-informed coping, mindfulness-based BP programs), and motivational interviewing to support self-efficacy; (iii) digital adherence support and home BP logs; (iv) targeted follow-up for women in the peri-/postmenopausal transition; and (v) nurse-led telemonitoring pathways to improve implementation where resources are limited. These steps align with the ESC’s emphasis on outcomes and implementation, targeting decisive and practical levers of BP control [5].

Regarding medication-induced BP elevation, the 2025 ESC Clinical Consensus specifically highlights medication-induced increases in BP among the secondary/iatrogenic contributors that must be systematically reviewed [5]. A practical one-minute checklist at each visit includes NSAIDs, estrogen-containing hormonal therapies (e.g., combined oral contraceptives), systemic corticosteroids, antidepressants with noradrenergic effects (e.g., SNRIs, TCAs) or MAOIs, psychostimulants/sympathomimetics (decongestants), calcineurin inhibitors (e.g., cyclosporine, tacrolimus), and anticancer/anti-rejection therapies known to raise BP. Where possible, discontinue or substitute the culprit; otherwise, intensify monitoring and adjust antihypertensive treatment. Including a systematic medication review improves diagnostic accuracy, prevents overtreatment, and aligns with ESC recommendations.

Among the spectrum of modifiable determinants of hypertension, psychological and behavioral factors, which include perceived stress, anxiety regulation, and self-efficacy, represent essential targets for intervention [121]. These factors can be effectively addressed through structured, evidence-based interventions that complement pharmacological and lifestyle approaches [122].

The treatment of psychological targets should co-occur with traditional physiological risk factor management, including obesity, dyslipidemia, and sedentary behavior, because these factors create reciprocal effects through neuroendocrine and behavioral mechanisms [123]. The low cost and preventive nature of psychological interventions, along with their easy scalability, make them suitable for resource-constrained healthcare settings [124,125].

Effective modalities include mindfulness-based stress reduction (MBSR), cognitive–behavioral therapy (CBT), motivational interviewing, and digital self-management programs [126,127]. These interventions improve adherence, resilience, and emotional regulation, indirectly enhancing the impact of standard medical and lifestyle care [127,128].

In terms of cost-effectiveness, psychological and behavioral programs are comparable to, or even better than, pharmacological intensification for mild-to-moderate hypertension, particularly when delivered through community or telemedicine channels [129,130]. Evidence from meta-analyses suggests that integrated psychosocial interventions can reduce systolic blood pressure by 4–8 mmHg—an effect magnitude similar to first-line antihypertensive monotherapy—at a fraction of the cost [131].

The primary beneficiaries are adults with newly diagnosed or uncontrolled hypertension. However, broader population-level initiatives (e.g., workplace stress management, community mindfulness training, or mobile health applications) extend benefits to normotensive individuals by reducing cardiovascular risk [132]. Integrating these approaches within multilevel prevention frameworks—combining public health promotion, digital tools, and personalized psychological care—offers a sustainable and equitable path to cardiovascular health [133].

## 5. Strengths and Limitations

A major strength of this study lies in its multidimensional assessment of psychological correlates of hypertension using several validated psychometric instruments. Clear group definitions and transparent reporting of effect sizes and confidence intervals enhance reproducibility and facilitate comparisons with international data. To our knowledge, this is among the first studies to examine these associations in a Romanian population, where hypertension prevalence and poor control rates remain among the highest in Europe.

Several limitations need to be considered in this study. The study design, which uses cross-sectional and correlational methods, prevents researchers from establishing cause-and-effect relationships; therefore, they should view observed associations as potential rather than causal. The research fails to establish whether stress, anxiety, and low self-efficacy function as factors that lead to hypertension, result from it, exist as independent variables, or create a two-way relationship with hypertension. The research data collection method based on self-reported information introduces potential biases, as participants may recall events inaccurately or present themselves in a more favorable light. The research design, which used online participation for mobility-restricted participants and family physician-based enrollment at offices, resulted in a more diverse participant group. The research design achieved greater inclusivity through its mixed recruitment approach. However, this method may have reduced the study’s representativeness, as online participants showed higher levels of health awareness and digital competence.

Another limitation concerns the absence of information on blood pressure control status among hypertensive participants (i.e., controlled versus uncontrolled hypertension). This unmeasured factor may moderate the observed psychological associations, as uncontrolled hypertension is frequently linked to higher stress and anxiety levels and diminished self-efficacy. Future studies should include direct assessments of blood pressure control and treatment adherence to better elucidate these relationships.

The post hoc power analysis showed that the study had enough power to detect medium effect sizes, but it might have missed small yet important differences. The study’s primary analyses had sufficient statistical power, but the researchers could not perform reliable subgroup analyses by socioeconomic status, education level, or sex. Future research needs to use bigger study populations that include different subgroups to study how different social factors affect the link between mental health issues and high blood pressure. The study lacks biological measures, including cortisol, catecholamines, and inflammatory cytokines, which prevents scientists from directly studying the mechanisms underlying the psychological–cardiovascular relationship.

The research employed independent-samples *t*-tests, Mann–Whitney U tests, and chi-square tests for descriptive and associative statistical analysis. The research methods used to identify group differences fail to establish causal or mechanistic connections among variables. Research scientists need to use longitudinal or experimental designs with randomized controlled trials to study psychological factors such as stress and self-efficacy while monitoring blood pressure changes over time.

## 6. Future Research Directions

For future research, it would be beneficial to build on these findings by using longitudinal cohort designs. This approach can help clarify the relationship between psychological distress and the onset of hypertension over time. By integrating biological and neuroimaging markers—such as cortisol levels, inflammatory cytokines, and patterns of activation in the hippocampus or prefrontal cortex—we can gain deeper insights into the underlying mechanisms. Additionally, conducting randomized controlled trials to evaluate various interventions could add valuable insights. This includes exploring the effectiveness of mindfulness-based programs, cognitive–behavioral therapy, mobile health platforms, and nurse-led telemonitoring on psychological well-being and blood pressure management. 

It is also relevant to address issues of health literacy and access to care, especially in parts of Eastern Europe with lower-income regions, where limited psychological resources and high cardiovascular risks often overlap. Expanding research to include populations with comorbid conditions, like diabetes and autoimmune diseases, may uncover shared pathways that contribute to a greater risk for cardiovascular issues. By taking these steps, we can work toward a more comprehensive understanding of how mental health and physical health interfere.

## 7. Conclusions

Hypertensive adults in our Romanian adult sample displayed a distinct psycho-emotional profile characterized by higher stress vulnerability and perceived stress, higher state and trait anxiety, and lower self-efficacy, whereas Type A behavior showed no association with HTN status. Although effect sizes were small to moderate, the consistency across instruments suggests that these psychological dimensions represent meaningful correlates of hypertension rather than incidental findings.

These results support the integration of psychosocial assessment into routine care for HTN. Brief, validated instruments such as the PSS-10, STAI, and GSES can identify at-risk patients, and targeted, evidence-based interventions—including mindfulness-based programs, cognitive–behavioral strategies, digital self-management tools, and nurse-led telemonitoring—may improve adherence, reduce distress, and contribute to better blood pressure control. Given the absence of a Type A signal, clinical attention should shift from fixed personality typologies to modifiable psychological and behavioral factors such as stress, anxiety, and self-efficacy.

It is important to note that the sample, while geographically diverse, was not nationally representative and included a predominance of women, likely reflecting sex differences in health-seeking behavior and postmenopausal vulnerability to hypertension. Consequently, the findings should be interpreted as preliminary and exploratory, offering region-specific insights rather than population-level generalizations. Future studies should employ larger and longitudinal designs incorporating biological mediators (e.g., HPA axis activity and inflammatory markers) to validate and develop these associations in nationally representative cohorts and culturally adapted cardiovascular care pathways.

## Figures and Tables

**Table 1:** Psychometric instruments used in the study.

Instrument	No. of Items	Scale Range	Subscales	Cronbach’s α (Present Sample)	Typical Range/Reference
**Stress Vulnerability Scale (SVS)**	20	1–5 (never–always)	—	0.87	0.82 [73]
**Perceived Stress Scale** **(PSS-10)**	10	0–4 (never–very often)	—	0.84	0.78–0.91 [74]
**State–Trait Anxiety Inventory (STAI-Y)**	40	1–4 (not at all–very much so)	STAI-S, STAI-T	0.91/0.89	>0.90 [75]
**General Self-Efficacy Scale (GSES)**	10	1–4 (not at all true–exactly true)	—	0.88	0.86–0.94 [76]
**Jenkins Activity Survey** **(JAS)**	52 (21 scored)	1–6	Type A Behavior Index	0.82	≈0.77 [77]

## Data Availability

The data presented in this study are available on request from the corresponding author.

## Supplementary Materials

The following supporting information can be downloaded at https://www.mdpi.com/article/10.3390/diseases13110373/s1. Table S1: Comparison between online- and office-recruited participants; Figure S1: Conceptual model linking hypertension, stress, anxiety, and self-efficacy.

## Abbreviations

The following abbreviations are used in this manuscript:

ESC	European Society of Cardiology
GSES	General Self-Efficacy Scale
HPA	The Hypothalamic–Pituitary–Adrenal Axis
HTN	Hypertension
JAS	Jenkins Activity Survey
LMICs	Low- and Middle-Income Countries
PSS-10	Perceived Stress Scale
ROS	Reactive Oxidative Species
SNS	Sympathetic Nervous System
STAI	State–Trait Anxiety Inventory
STAI-1	Trait Anxiety Inventory
STAI-2	State Anxiety Inventory
WHO	World Health Organization
