# Supplementary material for: Stress, Anxiety, and Self-Efficacy in Hypertension: Evidence from a Romanian Case—Control Study
Source: Diseases. 2025 Nov 13;13(11):373. doi: 10.3390/diseases13110373 (PMC12650833; doi:10.3390/diseases13110373)
Supplement: Supplementary file 1 [file diseases-13-00373-s001.zip › diseases-3941561-supplementary table S1.pdf]

Supplementary Table S1: Comparison between online- and office-recruited participants

| Variable | Online recruitment (n = 92) | Office recruitment (n = 123) | Statistical test | p-value |
|---|---|---|---|---|
| Age (years, mean ± SD) | 49.3 ± 10.7 | 51.1 ± 11.2 | t(213) = 1.12 | 0.26 |
| Gender (female, %) | 79.3% | 82.1% | $\chi^2(1) = 0.26$ | 0.61 |
| Education (university or higher, %) | 71.7% | 68.3% | $\chi^2(1) = 0.25$ | 0.62 |
| Place of residence (urban, %) | 89.1% | 62.6% | $\chi^2(1) = 15.84$ | <0.001 ** |
| Stress Vulnerability Scale (SVS, mean ± SD) | 43.8 ± 9.2 | 44.1 ± 8.8 | t(213) = 0.25 | 0.80 |
| Perceived Stress Scale (PSS-10, mean ± SD) | 18.4 ± 6.1 | 18.9 ± 6.4 | t(213) = 0.53 | 0.59 |
| State Anxiety (STAI-S, mean ± SD) | 44.7 ± 10.3 | 45.4 ± 10.7 | t(213) = 0.48 | 0.63 |
| Trait Anxiety (STAI-T, mean ± SD) | 45.2 ± 9.9 | 46.1 ± 10.1 | t(213) = 0.64 | 0.52 |
| General Self-Efficacy Scale (GSES, mean ± SD) | 30.1 ± 4.5 | 30.3 ± 4.3 | t(213) = 0.33 | 0.74 |
| Jenkins Activity Survey (JAS, Type A index) | 87.2 ± 10.9 | 88.0 ± 10.5 | t(213) = 0.52 | 0.60 |

Note: Values are presented as mean ± SD or percentages. Independent samples t-tests and Chi-square tests were used, as appropriate. No significant differences were found in psychological variables between recruitment modes. A higher proportion of urban participants were included in the online sample. ** $p < 0.001$.
