# Supplementary figures and images for: Stress, Anxiety, and Self-Efficacy in Hypertension: Evidence from a Romanian Case—Control Study
Source: Diseases. 2025 Nov 13;13(11):373. doi: 10.3390/diseases13110373 (PMC12650833; doi:10.3390/diseases13110373)

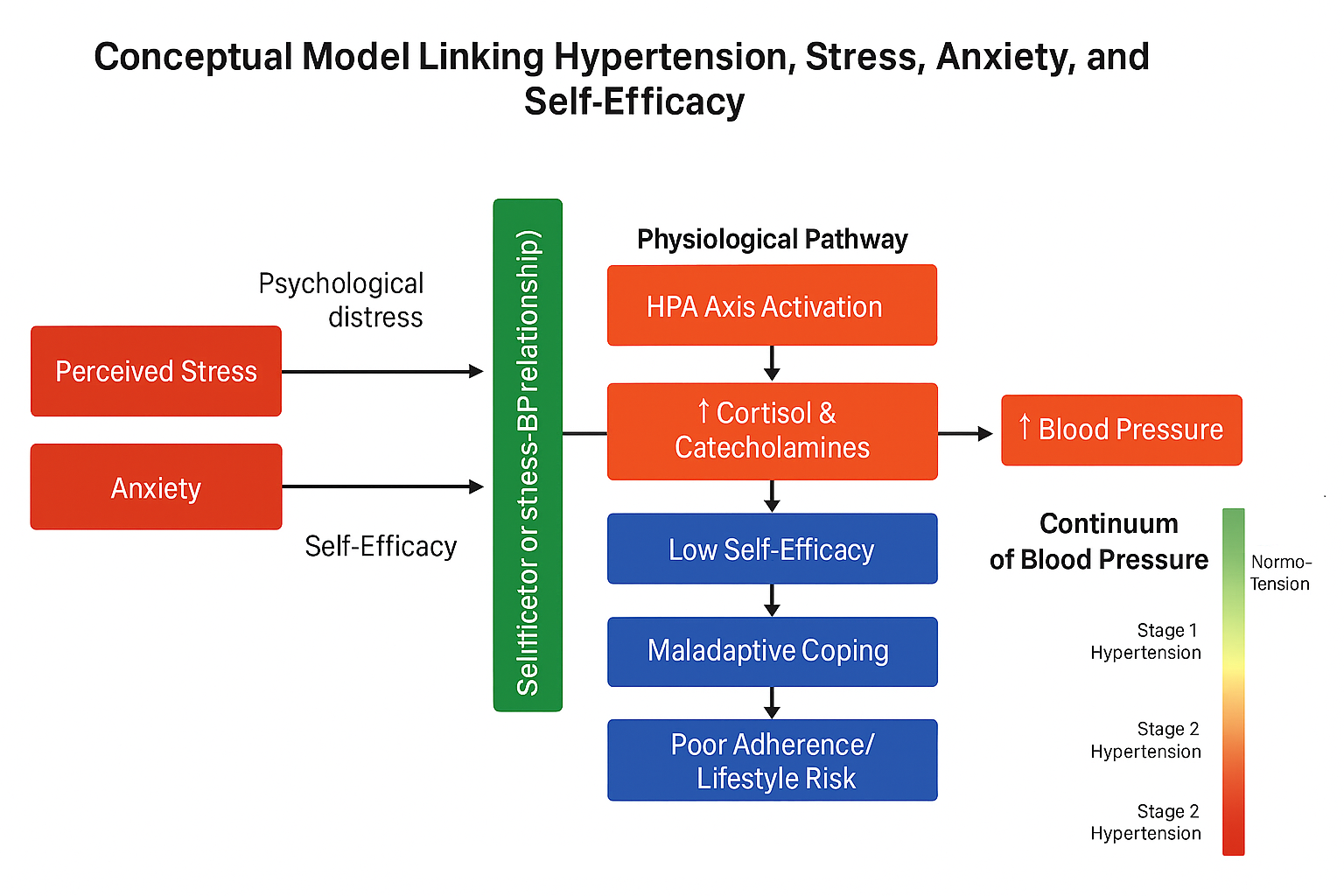

Supplement: Supplementary file 1 [file diseases-13-00373-s001.zip › diseases-3941561-supplementary figure S1.tif]
